# Trypanocidal Activity of *Smallanthus sonchifolius*: Identification of Active Sesquiterpene Lactones by Bioassay-Guided Fractionation

**DOI:** 10.1155/2013/627898

**Published:** 2013-06-06

**Authors:** F. M. Frank, J. Ulloa, S. I. Cazorla, G. Maravilla, E. L. Malchiodi, A. Grau, V. Martino, C. Catalán, L. V. Muschietti

**Affiliations:** ^1^Cátedra de Inmunología, IDEHU (UBA-CONICET), Facultad de Farmacia y Bioquímica Junín 956, 1113, Buenos Aires, Argentina, Instituto de Microbiología y Parasitología Médica, IMPaM (UBA-CONICET), Facultad de Medicina, Paraguay 215, 1121 Buenos Aires, Argentina; ^2^Cátedra de Farmacognosia, (IQUIMEFA) (UBA-CONICET), Facultad de Farmacia y Bioquímica, Junín 956, 1113 Buenos Aires, Argentina; ^3^Instituto de Ecología Regional (IER), Facultad de Ciencias Naturales, Universidad Nacional de Tucumán, 4107 Yerba Buena, Tucumán, Argentina; ^4^INQUINOA (CONICET), Facultad de Bioquímica, Química y Farmacia, UNT, Ayacucho 471, 4000 San Miguel de Tucumán, Argentina

## Abstract

In order to find novel plant-derived biologically active compounds against *Trypanosoma cruzi*, we isolated, from the organic extract of *Smallanthus sonchifolius*, the sesquiterpene lactones enhydrin (**1**), uvedalin (**2**), and polymatin B (**3**) by bioassay-guided fractionation technique. These compounds showed a significant trypanocidal activity against the epimastigote forms of the parasite with IC_50_ values of 0.84 **μ**M (**1**), 1.09 **μ**M (**2**), and 4.90 **μ**M (**3**). After a 24 h treatment with 10 **μ**g/mL of enhydrin or uvedalin, parasites were not able to recover their replication rate. Compounds **1** and **2** showed IC_50_ values of 33.4 **μ**M and 25.0 **μ**M against *T. cruzi* trypomastigotes, while polymatin B was not active. When the three compounds were tested against the intracellular forms of *T. cruzi*, they were able to inhibit the amastigote replication with IC_50_ of 5.17 **μ**M, 3.34 **μ**M, and 9.02 **μ**M for **1**, **2**, and **3**, respectively. The cytotoxicity of the compounds was evaluated in Vero cells obtaining CC_50_ values of 46.5 **μ**M (**1**), 46.8 **μ**M (**2**), and 147.3 **μ**M (**3**) and the selectivity index calculated. According to these results, enhydrin and uvedalin might have potentials as agents against Chagas disease and could serve as lead molecules to develop new drugs.

## 1. Introduction

Chagas disease, also called American trypanosomiasis is an endemic disease that remains as a major public health problem in Latin America. It is estimated that approximately 10 million people are infected and 100 million are at risk worldwide, mainly due to population migrations. The disease is caused by a kinetoplastid protozoan parasite, *Trypanosoma cruzi*, which is primarily transmitted by blood-sucking insects widely known in endemic countries as “kissing bugs.” The acute clinical stage of the disease (in which 5% of children die) is characterized by fever, generalized lymphadenopathy, and hepatosplenomegaly. The chronic stage often involves mainly cardiac and/or digestive disturbances being a leading cause of infectious cardiomyopathy worldwide [1].

Current treatments are based on the nitro-derivatives benznidazole and nifurtimox, two drugs developed more than four decades ago. They are employed in acute and early chronic cases. However, these drugs have an unsatisfactory cure rate in the chronic disease, are frequently not well tolerated, and cause toxic side effects [2]. Hence, improved treatment options are needed for all stages of *T. cruzi* infection. 

Medicinal plants produce a variety of chemical compounds and are still a major source of innovative therapeutic agents for various diseases, directly in their native form, or more often after optimization by structural modifications or by the synthesis of analogs with improved pharmacological properties [3]. Many reports concerning the antiprotozoal activity of natural compounds from plant origin have been reported [4–9]. 

*Smallanthus sonchifolius *(Asteraceae), a herbaceous perennial plant native to South America, is locally known as “yacon,” “llacuma,” and “jiquima” or “poire de terre” and “yacon strawberry” in Europe. The history of yacon goes far back beyond the Incas. This plant originates from the Andean region, whence it has spread to New Zealand, Japan, and Brazil. It is grown throughout the Andes, from Colombia to Northwestern Argentina [10, 11], and its cultivation and consumption have expanded in recent decades to several Asian and European countries [12]. Yacon has been identified as a traditional food in the Andean region, and it is also used as an offering during religious festivities [11]. Besides from its use as food, yacon is also acknowledged as a medicinal plant. Antidiabetic properties have been attributed to yacon leaves, which are dried and used in the preparation of tea [12]. Previous biological works have demonstrated anti-inflammatory, antifungal, and antibacterial activities [13, 14]. Different chemical compounds such as phenolic and ent-kaurenoic acids, related diterpenoid substances, acetophenone phytoalexins, and sesquiterpene lactones (STLs) were identified in yacon leaves [15, 16]. 

We have already shown that STLs, mainly occurring in the Asteraceae family, represent a class of compounds with potential as trypanocidal leads [17–19]. Given the increasing interest that yacon has raised in recent years due to its health promoting properties, its potential as a medicinal plant, and its content in STLs, we have selected *S. sonchifolius* in the search for trypanocidal molecules against *T. cruzi.*

## 2. Materials and Methods

### 2.1. Plant Material

Leaves of *Smallanthus sonchifolius* (Poepp. & Endl.) H. Robinson (Asteraceae), clone LIEY 97-2, were collected in May 2010 from experimental crops located at Centro Universitario “Horco Molle,” Universidad Nacional de Tucumán (26°47′ S, 65°19′ W, 547 m a.s.l.). A voucher specimen was deposited at the Herbarium of Instituto Miguel Lillo, S. M. de Tucumán, Argentina (LIL 607176).

### 2.2. Extraction of Plant Material

Approximately 350 g of air-dried and ground leaves were extracted by soaking in dichloromethane (6.8 L) at room temperature for 30 min. The procedure was repeated, and the filtrates combined. Evaporation of the solvent under reduced pressure provided 9.29 g of organic extract (OE; 2.65%).

### 2.3. Bioassay-Guided Fractionation of OE and Isolation of Compounds

The OE (9.0 g) was separated by column chromatography (CC) on silica gel 60 (Merck, 0.063–0.2 mm/70–230 mesh; 67 g) affording 10 fractions (*F*_1A_–*F*_5B_). Each fraction was eluted with 250 mL of the following eluents: *n*-hexane 100% (*F*_1A_-*F*_1B_), *n*-hexane : ethyl acetate 1 : 1 (*F*_2A_-*F*_2B_.), ethyl acetate 100% (*F*_3A_-*F*_3B_), ethyl acetate : methanol 1 : 1 (*F*_4A_-*F*_4B_), and methanol 100% (*F*_5A_-*F*_5B_). Eluates were monitored by thin-layer chromatography (TLC) using silica gel 60 *F*_254_ (Merck) using hexane : ethyl acetate 1 : 1 as mobile phase. Visualization of compounds was done by a solution of anisaldehyde/sulfuric acid followed by heating. 

Fractions *F*_2B_ and *F*_3A_ were separately chromatographed over silica gel CC (10 g) and eluted isocratically with *n*-hexane : ethyl acetate (1 : 1) as mobile phase yielding 20 subfractions of *F*_2B_ (*F*_2B1_–*F*_2B20_) and 22 subfractions from *F*_3A_ (*F*_3A1_–*F*_3A22_), of 10 mL each. According to their TLC profile, fractions *F*_2B5_–*F*_2B7_; *F*_2B8_–*F*_2B12_; *F*_3A6_–*F*_3A8_ and *F*_3A9_–*F*_3A13_ were pooled and subjected to preparative TLC on silica gel 60 employing *n*-hexane : ethyl acetate 1 : 1 as mobile phase to afford compounds **1**, **2**, and **3**. 

These compounds were recrystallized from ethanol at 96°C and identified by spectroscopic techniques: ^1^H- and ^13^C-nuclear magnetic resonance (NMR), gas chromatography coupled to mass spectrometry (GC/MS), and infrared spectroscopy (IR). 

### 2.4. Identification of Compounds **1**, **2,** and **3**

 The structure elucidation of the isolated compounds was performed by proton nuclear magnetic resonance (^1^H NMR) and carbon NMR (^13^C NMR) (Bruker 300 MHz-Karlsruhe, Germany). Compounds were dissolved in deuterated chloroform (Cl_3_CD), and tetramethylsilane (TMS) was used as internal standard (Sigma). FTIR spectra were recorded on a Nicolet 380 spectrometer using KBr pellets. The material was dried and placed in a desiccator at 20°C prior to pellet preparation. Gas chromatography/mass spectroscopy spectra were recorded on a Agilent 5973 network mass selective detector (MS), Agilent 6890 Series GC system (GC).

### 2.5. Parasites, Cell Lines, and Media

*T. cruzi* epimastigotes (RA strain) were grown in liver infusion tryptose medium (LIT) supplemented with 10% fetal bovine serum (Natocor). Cultures were routinely maintained by weekly passages at 28°C. *T. cruzi* trypomastigotes were routinely maintained by infecting 21-day-old male CF1 mice.* T. cruzi* amastigotes were obtained by infecting J774 cells with bloodstream trypomastigotes.

### 2.6. *In Vitro* Trypanocidal Activity against *T. cruzi* Epimastigotes and Trypomastigotes

*S. sonchifolius* OE and fractions *F*_1A_–*F*_5B_ were tested for trypanocidal activity against *T. cruzi *epimastigotes, at final concentrations of 10 *μ*g/mL and 100 *μ*g/mL, by a [^3^H] thymidine uptake assay as previously described [17]. Compounds **1**, **2**, and **3** were tested at final concentrations ranging from 0.01 *μ*g/mL to 50 *μ*g/mL. Stock solutions of the samples were prepared in ethanol : water (1 : 1). Epimastigotes in exponential growth phase were adjusted to 1.5 × 10^6^ parasites/mL and seeded on 96-well plates in the presence of the different concentrations of OE, fractions, or compounds. Parasites were cultured in triplicate for 72 h. Control parasites were cultured in absence or presence of benznidazole (20 *μ*M; Roche-Rio de Janeiro, Brazil). Percentage inhibition was calculated as 100 − {[(cpm of treated parasites)/(cpm of untreated parasites)] × 100}. The compound concentration at which the parasite growth was inhibited by 50% (inhibitory concentration 50, IC_50_) was determined after 72 h.

To determine whether the parasites could recover after treatment with the pure compounds, *T. cruzi* epimastigotes were incubated with compounds **1**–**3** (0.1, 1, and 10 *μ*g/mL) for 24 h. Parasites were then centrifuged at 3000 rpm for 10 min, resuspended in fresh medium, and incubated for 2 additional days, counting the number of parasite in a Neubauer chamber on a daily basis.

The trypanocidal effect of compounds **1**, **2**, and **3** was also tested on bloodstream trypomastigotes as previously described [17]. Briefly, mouse blood containing trypomastigotes was adjusted to a concentration of 1.5 × 10^6^ parasite/mL and seeded (150 *μ*L/well) by duplicate into a 96-well microplate, in the presence of each compound (1 to 50 *μ*g/mL, final concentration). Plates were incubated for 24 h, and the remaining live parasites were counted in a haemocytometer.

### 2.7. *In Vitro* Trypanocidal Activity against *T. cruzi* Amastigotes

To evaluate the effect of compounds **1–3** on intracellular forms of *T. cruzi*, 96-well plates were seeded with J774 murine macrophages at 5 × 10^3^ per well in 100 *μ*L of culture medium and incubated for 2 h at 37°C in a 5% CO_2_ atmosphere. Cells were infected with transfected blood trypomastigotes expressing *β*-galactosidase at a parasite : cell ratio of 10 : 1. After 2 h of coculture, plates were washed twice with PBS to remove free parasites and compounds **1–3** were added at 0.1–50 *μ*g/mL per well in 150 *μ*L of fresh complete RPMI medium without phenol red (Gibco, Rockville, MD). Controls included infected nontreated cells (100% infection control) and uninfected cells (0% infection control). The assay was developed 48 h later by addition of chlorophenol red-*β*-d-galactopyranoside (100 *μ*M CPRG) and 1% Nonidet P40. Plates were incubated for 4–6 h at 37°C. Wells with galactosidase activity turned the media from yellow to red, and this reaction was quantified at 590 nm in a microplate reader (Bio-Rad Laboratories, Hercules, CA). Percentage inhibition was calculated as 100 − {[(absorbance of treated infected cells)/(absorbance of untreated infected cells)] × 100} and the IC_50_ value calculated.

### 2.8. Cytotoxicity Assay

The cytotoxic effect of compounds **1**, **2**, and **3** on Vero cells was evaluated by using the MTT tetrazolium salt (3-(4,5-dimethylthiazol-2-yl)-2,5-diphenyltetrazolium bromide) (Sigma) colorimetric assay. Cells (5 × 10^4^ cells/well) were seeded at a final volume of 150 *μ*L in a flat-bottom 96-well microplate and cultured at 37°C in a 5% CO_2_ atmosphere in the absence or presence of increasing concentrations of the compounds (1–50 *μ*g/mL). After 24 h, MTT was added at a final concentration of 1.5 mg/mL and plates were incubated for 2 h at 37°C. The purple formazan crystals were completely dissolved by adding 150 *μ*L of ethanol, and the absorbance was detected at 570 nm in a microplate reader. Results were calculated as the ratio between optical density in the presence and absence of the compound multiplied by 100. The 50% cytotoxic concentration (CC_50_) was calculated for each compound. All experiments were made in duplicate.

The selectivity index (SI) of each compound was calculated as the CC_50_ obtained with Vero cells divided by the IC_50_ obtained against *T. cruzi* amastigotes.

### 2.9. Statistical Analysis

All values were presented as mean ± SEM The GraphPad Prism 3.0 software (GraphPad Software Inc., San Diego, CA) was employed to carry out calculations. To calculate the IC_50_ values, the percentages of inhibition were plotted against the drug concentration and fitted with a straight line determined by a linear regression (Sigma Plot 10 software). Results presented are representative of three to four independent experiments.

## 3. Results

### 3.1. *In Vitro* Trypanocidal Activity of *S. sonchifolius* OE

The trypanocidal activity of the organic extract of *S. sonchifolius* (OE) was evaluated *in vitro* against *T. cruzi* epimastigotes by a [^3^H] thymidine uptake assay. The extract was found to be active showing a growth inhibition of 87.6% ± 5.3 and 95.1% ± 0.5 at 100 and 10 *μ*g/mL, respectively.

**Figure 1:**
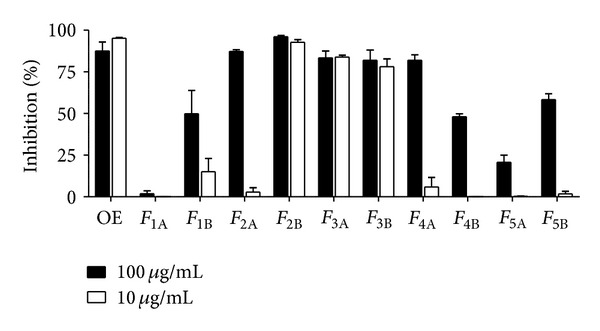
Growth inhibition of* T. cruzi* epimastigotes by *S. sonchifolius* OE and fractions *F*_1A_ to *F*_5B_. Epimastigotes were cultured in triplicate in the presence of 10 or 100 *µ*g/mL of OE or each fraction. Cultures were done in 96-well plates with 1.5 × 10^6^ parasites/mL during 72 h with the addition of [^3^H] thymidine for the last 16 h. Bars represent means ± SEM.

The fractionation of the OE by CC yielded 10 fractions (*F*_1A_–*F*_5B_), the effects of which were tested against epimastigote forms of *T. cruzi* as described previously (Figure 1). The results showed that at the lowest concentration tested (10 *μ*g/mL) fractions *F*_2B_, *F*_3A_, and *F*_3B_ showed the highest trypanocidal activity with percentages of growth inhibition of 92.7 ± 1.7%, 83.8 ± 1.1%, and 82.7 ± 1.0%, respectively. 

### 3.2. Bioassay-Guided Fractionation of *S. sonchifolius* OE

**Figure 2:**
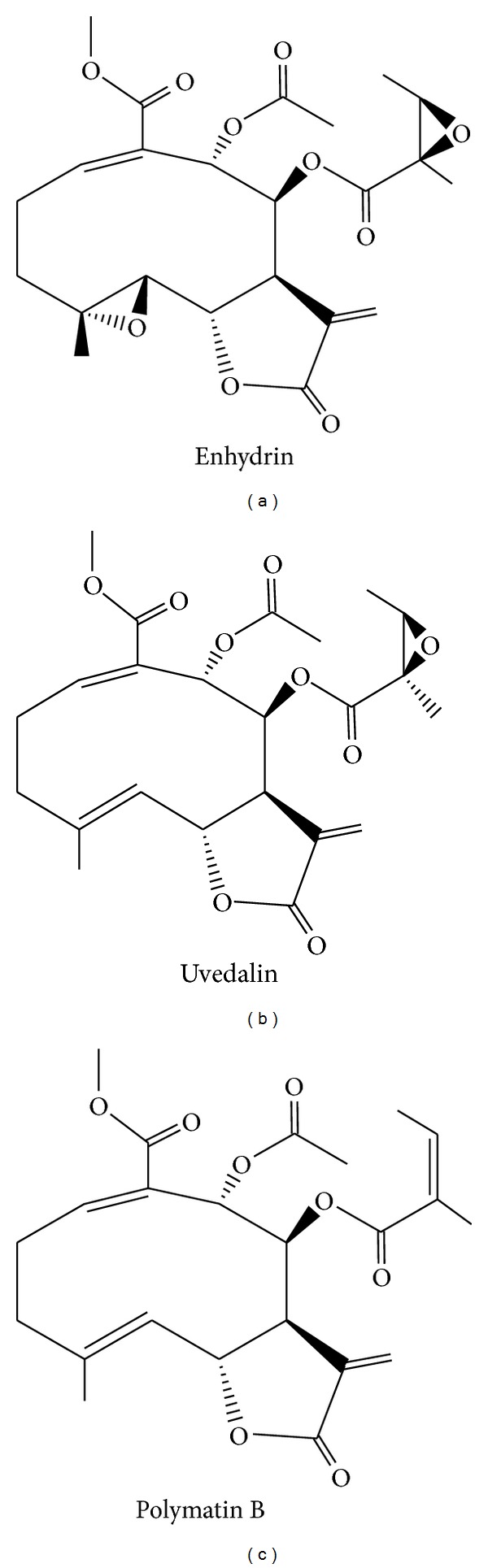
Chemical structures of the sesquiterpene lactones enhydrin, uvedalin, and polymatin B.

The bioassay-guided fractionation of fractions *F*_2B_ and *F*_3A_ led to the isolation of compounds **1**, **2**, and **3** that were purified by a combination of column chromatography, preparative TLC, and precipitation techniques. Compound **1** (35 mg; 0.39%), compound **2 **(6.4 mg; 0.07%), and compound **3** (2.4 mg; 0.03%) were identified by comparing their spectral data with published values [20–22] as the STLs *enhydrin* (4*α*,5*β*-epoxy-8*β*-(2′S,3′S)-epoxyangeloyloxy-9*α*-acetyloxy-1(10)E,11(13)-germacradien-12,6*α*-olide-14-oic-acid methyl ester), *uvedalin* (8*β*-(2′S,3′S)-epoxyangeloyloxy-9*α*-acetyloxy-1(10)E,4E,11(13)-germacratrien-12,6*α*-olide-14-oic acid methyl ester), and *polymatin B* (8*β*-angeloyloxi-9*α*-acetyloxi-1(10)E,4E,11(13)-germacratrien-12,6*α*-olide-14-oic acid methyl ester) (Figure 2). Purity of the compounds (>95%) was confirmed by gas chromatography (GC).

### 3.3. *In Vitro* Trypanocidal Activity against *T. cruzi* Epimastigotes and Trypomastigotes

**Figure 3:**
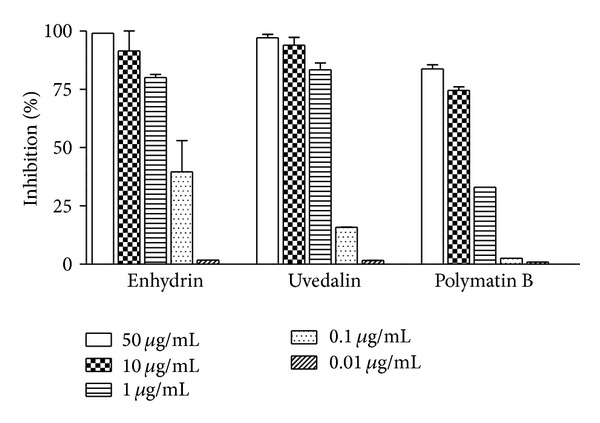
Inhibition of *T. cruzi* epimastigotes growth by enhydrin, uvedalin, and polymatin B. Parasites were adjusted at 1.5 × 10^6^/mL and incubated in triplicate in the presence of 0.01 to 50 *μ*g/mL of each compound. Parasites were cultured for 72 h, with the addition of [^3^H] thymidine for the last 16 h.

The IC_50_ values of the pure compounds were calculated on different *T. cruzi* evolutive stages.  Figure 3 shows the effect of the pure compounds on the growth of epimastigotes of *T. cruzi*. The IC_50_ values for enhydrin, uvedalin, and polymatin B were 0.39 *μ*g/mL (0.84 *μ*M), 0.49 *μ*g/mL (1.09 *μ*M), and 2.12 *μ*g/mL (4.90 *μ*M), respectively, after 72 h of incubation.

**Figure 4:**
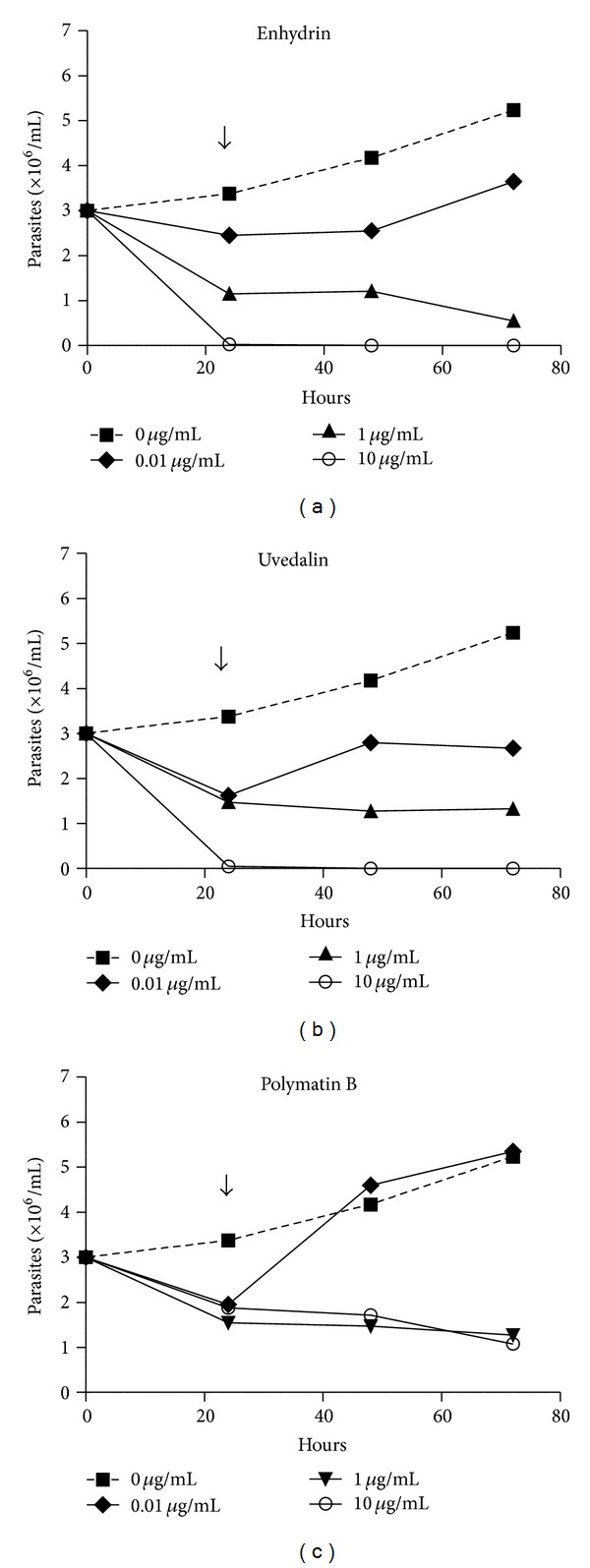
Residual effect of enhydrin, uvedalin, and polymatin B on the growth of *T. cruzi* epimastigotes. Parasites were incubated in the absence or presence of 0–10 *μ*g/mL of the compounds for 24 h. The culture medium was replaced by a fresh one (arrow) without the compound, and parasites were allowed to grow for 2 days. Parasites were counted in a Neubauer chamber. Symbols represent the mean ± SEM.

Moreover, after the 24 h treatment with 10 *μ*g/mL enhydrin or uvedalin, a drastic reduction in the amount of parasites could be observed. Besides, two days after removal of the compounds, no recuperation of the epimastigotes was observed suggesting that 24 h treatment at high doses is sufficient to kill the parasites (Figure 4). When 0.1 *μ*g/mL of polymatin B was removed at 24 h after treatment, epimastigotes recovered their replication rates at values similar to the control parasites.

**Figure 5:**
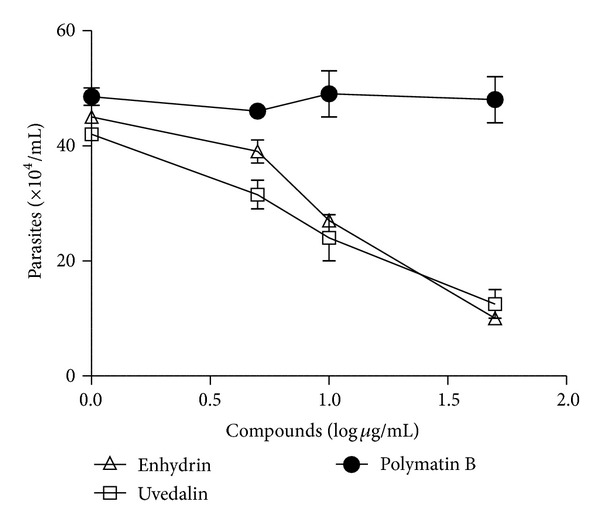
Effect of enhydrin, uvedalin, and polymatin B on *T. cruzi* trypomastigotes. Bloodstream trypomastigotes were cultured in duplicate in the presence of 1 to 50 *µ*g/mL of the compounds. The assay was performed employing 1.5 × 10^6^ parasites/mL over 24 h. Remaining live parasites were counted in a Neubauer chamber. Symbols represent the mean ± SEM.

The effect of the compounds against the infective form of *T. cruzi* is shown in Figure 5. When bloodstream trypomastigotes were incubated with the pure STLs, we observed that enhydrin and uvedalin were active with IC_50_ values of 15.5 *μ*g/mL (33.4 *μ*M) and 11.2 *μ*g/mL (25.0 *μ*M), respectively. By contrast, polymatin B showed no activity against this parasite form.

### 3.4. *In Vitro* Trypanocidal Activity against *T. cruzi* Amastigotes

**Figure 6:**
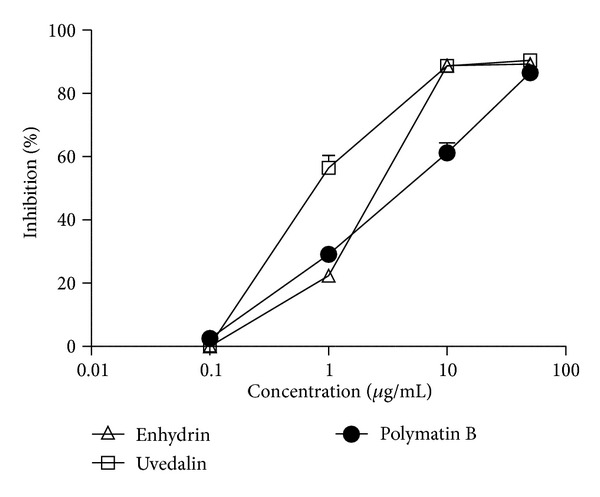
Effect of enhydrin, uvedalin, and polymatin B on *T. cruzi *amastigotes. J774 phagocytic cells were infected with transfected trypomastigotes expressing *β*-galactosidase (10 : 1 parasite : cell ratio). After removing free parasites, STLs were added at concentrations ranging from 0.1 to 50 *μ*g/mL. Three days after infection, nonidet P40 and chlorophenol red-*β*-d-galactopyranoside (CPRG) were added and the galactosidase activity was determined at 590 nm. Values represent the mean ± SEM.

In order to evaluate the ability of the pure compounds to inhibit the intracellular amastigote forms of *T. cruzi*, J774 macrophages were infected with transfected blood trypomastigotes expressing *β*-galactosidase. Forty-eight hours after the addition of the pure compounds, the percentage of inhibition was determined. All the tested STLs were able to inhibit amastigotes replication with IC_50_ values of 2.4, 1.5, and 3.9 *μ*g/mL (5.17, 3.34, and 9.02 *μ*M) for enhydrin, uvedalin, and polymatin B, respectively (Figure 6).

### 3.5. Cytotoxicity Activity in Vero Cells

**Figure 7:**
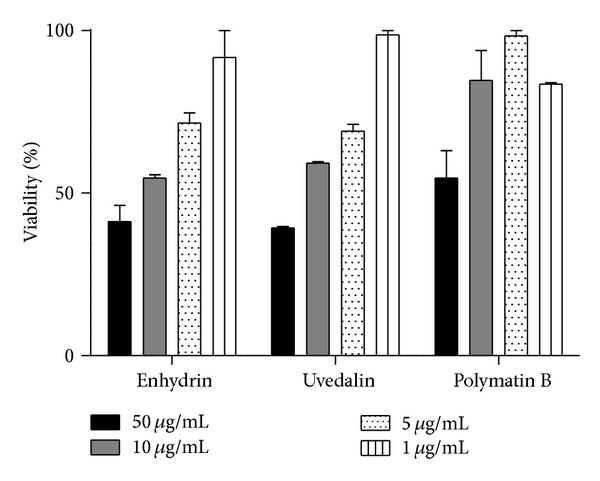
Cytotoxicity of enhydrin, uvedalin, and polymatin B on Vero cells. Cultures were kept for 24 h in the presence of different concentrations (1 to 50 *μ*g/mL) of the STLs. Cell viability was determined by the MTT method and was expressed as the ratio between viable cells in the presence and absence of the compound multiplied by 100. Bars represent the mean ± SEM of three experiments carried out in duplicate.

*In vitro* cytotoxicity of the STLs on Vero cells was analyzed using the MTT assay. Results are shown in Figure 7. When cell suspensions were treated with enhydrin, uvedalin, and polymatin B, the CC_50_ were 21.6, 21.0, and 63.7 *μ*g/mL (46.5, 46.8, and 147.3 *μ*M), respectively. The SI was used to compare the toxicity for mammalian cells and the activity against the parasites. The SI for the intracellular form of the parasites was 9, 14, and 16.3 for enhydrin, uvedalin, and polymatin B, respectively. 

## 4. Discussion

In this work, the trypanocidal activity of the species *Smallanthus sonchifolius* has been evaluated by *in vitro *assays. The dichloromethane extract (OE) of this plant induced a significant growth inhibition (95.1%) when tested against *T. cruzi* epimastigotes at a concentration of 10 *μ*g/mL. This result prompted us to carry out a bioassay-guided fractionation of the OE by chromatographic techniques. Among the tested fractions, *F*_2B_, *F*_3A_, and *F*_3B_ presented the highest *in vitro* inhibitory activity, against epimastigotes, with percentages of growth inhibition higher than 80% at the lower concentration tested. Further purification of *F*_2B_ and *F*_3A_, by a series of chromatographic separations, led to the isolation of three structurally related germacranolide STLs of the melampolide type, which were identified as enhydrin, uvedalin, and polymatin B. 

STLs are naturally occurring plant terpenoids with over 5000 known structures and which are mainly present in members of the Asteraceae family [23]. They exhibit a variety of skeletal arrangements and are the largest and most diverse category of natural products with an *α*-methylene-*γ*-lactone motif [3]. STLs have been related to a broad spectrum of biological activities ranging from anticancer, antiviral, antibacterial, antifungal, and antiprotozoal. Many are described as the active constituents of medicinal plants used in traditional medicine for the treatment of inflammatory diseases [24]. The vast majority of STLs are considered quite “drug-like” molecules with respect to their physicochemical properties [25]. 

Enhydrin, uvedalin, and polymatin B were firstly evaluated for their trypanocidal activity on *T. cruzi* epimastigotes showing a marked activity with IC_50_ values of 0.84 *μ*M, 1.09 *μ*M, and 4.90 *μ*M, respectively. These values indicated that an increase in the trypanocidal activity was achieved along the purification process.

Besides, epimastigotes treated for 24 h with 10 *μ*g/mL of enhydrin or uvedalin were not able to recover their initial replication rates suggesting that the treatment at high doses was sufficient to kill the parasites. Parasites treated with either enhydrin or uvedalin, at 1 *μ*g/mL, or with polymatin B, at 10 *μ*g/mL, presented a reduction in the replication rate that was close to 50% at 24 h after treatment. On the other hand, recovery was evident when 0.1 *μ*g/mL of the compounds was employed. 

When the isolated compounds were evaluated against *T. cruzi *trypomastigotes, the results (IC_50_ = 33.4 *μ*M for enhydrin and IC_50_ = 25.0 *μ*M for uvedalin) showed that this parasite stage is less sensitive than epimastigotes to the compounds. During the initial acute phase of infection, the nonreplicative bloodstream trypomastigotes invade different mammalian cell types, where they transform into replicative intracellular amastigotes and multiply within the host's cells cytoplasm. Interestingly, when enhydrin, uvedalin, and polymatin B were tested against the amastigote forms, they were able to inhibit replication. The three STLs were active with IC_50_ values of 5.17 *μ*M, 3.34 *μ*M, and 9.02 *μ*M, respectively. The ability of compounds to inhibit the intracellular growth of *T. cruzi* amastigotes is a more rigorous and relevant test of anti-*T. cruzi* activity, as it is applied to a stage which is the predominant form in mammals and because the killing assay requires that the drug cross the host cell membrane [26]. It is well known that the treatment with benznidazole is especially useful for patients in the acute phase, when trypomastigotes may be easily found in blood, while its effectiveness during the asymptomatic or chronic stage, is still controversial. The fact that these compounds proved to be active against amastigotes is of particular interest, since the DNDi organization prioritizes the development of drugs that are useful during the indeterminate and chronic phases of the infection, where parasites remain intracellular [1]. 

The therapeutic potential and the lack of cytotoxic effects on mammalian cells are important criteria to be considered when novel compounds with activity against *T. cruzi *are investigated. Compounds containing an *exo*methylene moiety conjugated with a carbonyl group can react as Michael-type acceptors with the thiol groups of macromolecules such as enzymes [3]. The presence of this reactive group in the isolated STLs could explain the trypanocidal effects and the cytotoxicity of these compounds. When analyzing the chemical structure of the STLs isolated in this work, it could be observed that, unlike enhydrin and uvedalin, polymatin B does not present an epoxide as substituent. Biological activity of *α*-methylene-*γ*-lactones may vary according to their different number of alkylating structure elements such as conjugated cyclopentones, conjugated side-chain esters, and epoxides [25]. These features may be related to the increased activity shown by enhydrin and uvedalin compared to the nonepoxide substituted polymatin B. Moreover, when comparing the toxicity of the lactones in Vero cells with the activity against *T. cruzi* amastigotes, we could observe that enhydrin, uvedalin, and polymatin B had some selective toxicity against the parasites (SI of 9, 14, and 16.3, resp.). 

The three STLs had been previously isolated from *S. sonchifolius* [11, 13, 15]. Enhydrin and uvedalin have shown cytotoxic activity in cervical cancer cells [27]. For enhydrin hypoglycemic, antibacterial, anti-inflammatory, and antihyperalgesic activities have also been reported [11, 28, 29], while this is the first report of the anti *T. cruzi* activity of these compounds. 

## 5. Conclusion 

Three STLs with promising antitrypanocidal properties were isolated from the leaves of *S. sonchifolius* by a bioassay-guided fractionation method. The compounds, identified as enhydrin, uvedalin, and polymatin B, efficiently inhibited both the epimastigote and the replicative intracellular amastigotes, being more selective for the parasites than for mammalian cells. According to the results obtained, enhydrin and uvedalin may be considered promising antitrypanosomal lead molecules. However, the potential of these compounds still have to be improved by obtaining derivatives that might be used as therapeutic agents against the parasite. 

Further studies will involve evaluation of the underlying mechanisms as well as *in vivo* studies of enhydrin and uvedalin.
